# Progressive Pseudorheumatoid Dysplasia or JIA?

**DOI:** 10.1155/2017/1609247

**Published:** 2017-02-21

**Authors:** Geetha Wickrematilake

**Affiliations:** District General Hospital, Nuwara Eliya, Sri Lanka

## Abstract

Progressive pseudorheumatoid dysplasia (PPD) or spondyloepiphyseal dysplasia tarda with progressive arthropathy (SEDT-PA) is a rare arthropathy of childhood involving the axial skeleton as well as small peripheral joints. A 10-year-old boy was referred by a general practitioner with pain and deformity in the fingers of hands and limping gait. There was no joint synovitis although the finger joints were bulky on examination with mild flexion deformity. Patient had exaggerated kyphosis and lumbar lordosis with pigeon chest and restricted hip joint movements. Anteroposterior X-rays of the hip joints revealed widened and flattened epiphyses of the femoral heads with narrow and irregular joint spaces. Hand X-rays revealed periarticular osteopenia, significant narrowing of the joint spaces of proximal interphalangeal, and distal interphalangeal joints, together with osseous enlargement of the basis of metacarpal bones and phalanges. Spinal X-rays revealed generalized platyspondyly and anterior beaking of vertebral bodies. There was a clear mega os trigonum in his feet images. All blood investigations were normal with no evidence of inflammation and thyroid hormone levels were normal. The diagnosis of PPD was favored by imaging studies and normal inflammatory markers and the patient was treated with physiotherapy, family counseling, and anti-inflammatory medications.

## 1. Introduction

Spondyloepiphyseal dysplasia tarda with progressive arthropathy (SEDT-PA) is a rare hereditary disorder with autosomal recessive inheritance [1]. This primarily affects the articular cartilage [2].

It is characterized by short trunk and extremities, platyspondyly, kyphoscoliosis, coxa vara, genu valgum/varum, and abnormal shape and structure of the epiphyses of hand bones. With time, this leads to disproportionate dwarfism with progressive involvement of the spine and epiphysis of long bones. Boys are affected more often [3, 4].

Since joint involvement in SEDT-PA may be confused with juvenile idiopathic arthritis (JIA), SEDT-PA is also called progressive pseudorheumatoid dysplasia of childhood (PPD) [5, 6].

It is a rare disorder with a global prevalence of only 1/1 000 000 with two-thirds of reported cases from Arabia [7].

## 2. Patient Information and Clinical Findings

The patient was an intelligent 10-year-old boy born at term after a normal pregnancy and delivery. His parents were not consanguineous and did not have any family history of joint problems although both parents had Arabic origins.

His birth weight, neonatal history, and developmental milestones were normal. His symptoms had started at the age of 7 years with pain in the right hip with limping. This was followed by pain in left hip with swelling and deformities in the hands, wrists, and elbows.

His condition was misdiagnosed by several medical practitioners and was treated as JIA with nonsteroidal anti-inflammatory medications. However, luckily the patient was not started on disease modifying antirheumatic drugs. His height on presentation was <3rd percentile and upper- to lower-body segment ratio was 0.83. On examination his hands showed bulky proximal and distal interphalangeal joints without any synovitis. There was mild fixed flexion deformity of fingers. Neurological examination was uneventful.

## 3. Diagnostic Assessment 

**Figure 1:**
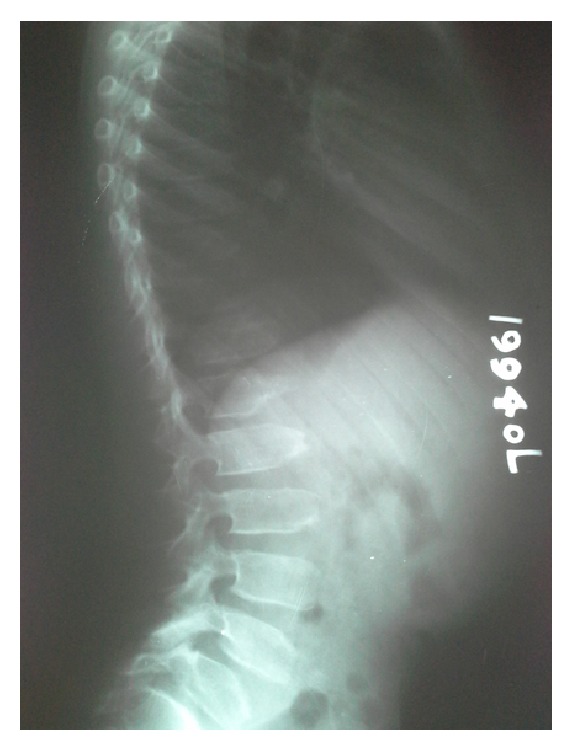
Platyspondyly with and anterior end plate abnormalities with defective ossification and anterior beaking of vertebral bodies.

**Figure 2:**
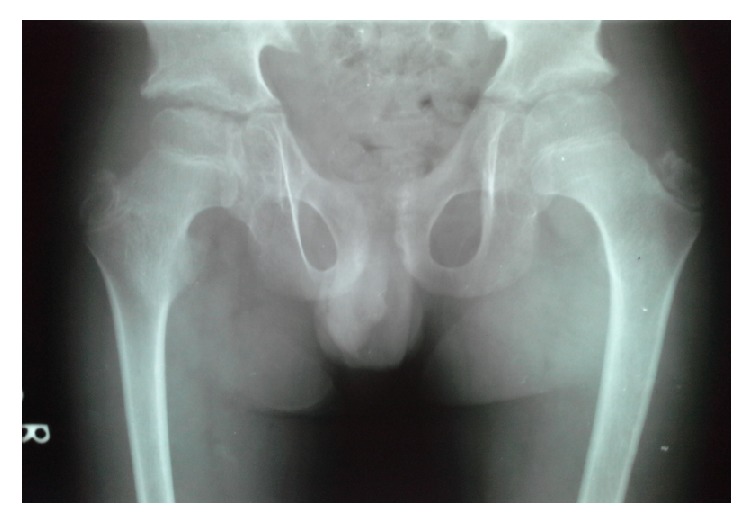
Anteroposterior X-ray of the pelvis showing flattened and enlarged epiphyses of the femoral heads.

Radiological examination showed platyspondyly with anterior beaking of vertebral bodies (Figure 1), irregular enlarged femoral epiphyses with narrowing of the hip joint spaces, premature osteoarthritis, and short femoral necks (Figure 2).

**Figure 3:**
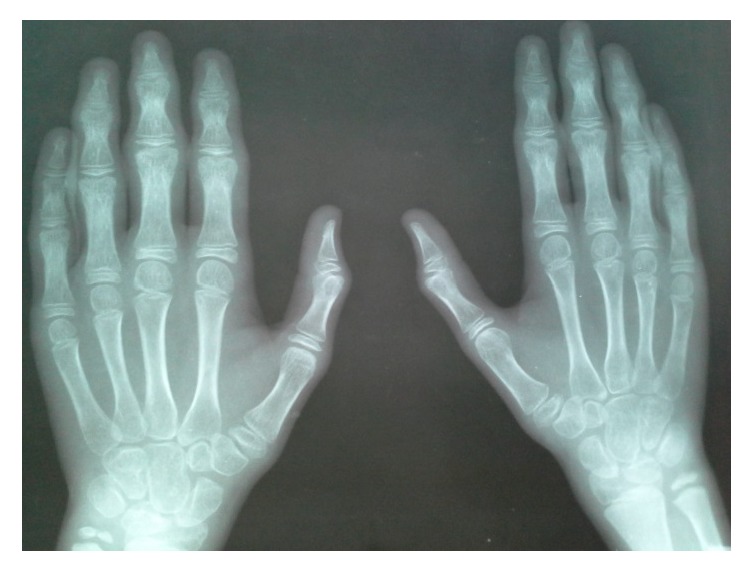
X-ray of hands showing epiphyseal enlargement of the metacarpophalangeal and interphalangeal joints with juxta-articular osteopenia.

**Figure 4:**
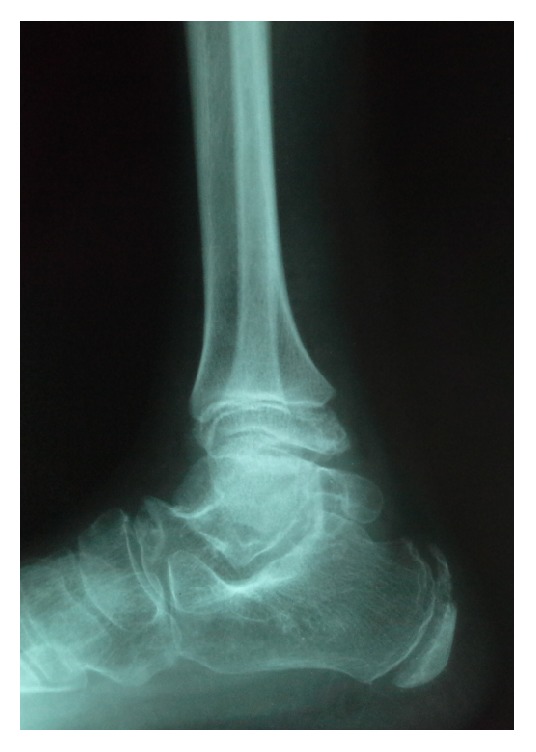
Lateral radiograph of right foot indicating mega os trigonum.

The phalangeal epiphyses and metaphyses were enlarged and osteoarthritic changes were present in the interphalangeal joints (Figure 3). Enlarged os trigonum was seen in foot X-rays (Figure 4).

Hematological investigations including serum calcium/phosphate, thyroid hormones, full blood count, C reactive protein, erythrocyte sedimentation rate, rheumatoid factor, and antinuclear antibody levels were all negative. Mucopolysaccharides were negative in the urine with normal skull imaging.

## 4. Therapeutic Intervention and Follow-Up

The child was clinically having short stature with kyphoscoliosis, pigeon chest, and joint problems. X-ray images of the pelvis show irregular ossification of the heads and greater trochanters of the femora and the X-rays of spine showed generalized platyspondyly with anterior beaking. Considering these facts, differential diagnosis like Faber's disease, Morquio syndrome, Scheuermann's disease, and JIA should be ruled out.

A diagnosis of SEDT-PA was made with patients history, examination, and X-ray findings and the condition was explained to the family. Patient was treated with physiotherapy and was referred to the orthopedic surgeon for assessment and follow-up as he would need corrective surgery in future. Ophthalmological opinion was taken to exclude any eye involvement. Family was referred for genetic counseling.

Physiotherapy was arranged and patient was prescribed nonsteroidal anti-inflammatory medications to take when needed for joint pains.

## 5. Discussion

PPD disease generally begins between the ages of 2 and 8 years with abnormal gait and fatigability [8]. The differentiation between JIA and SEDT-PA is challenging and most cases are misdiagnosed in the beginning as JIA. However, the absence of laboratory changes indicating systemic or synovial inflammation and the presence of characteristic X-ray changes of spondyloepiphyseal dysplasia help in the differential diagnosis of SEDT-PA from JIA [9].

The main reasons for misdiagnosis appear to be onset in childhood with swelling and restriction of peripheral and axial joints and the rarity of the disease.

The main clinical features in PPD are arthralgia, joint contractures, enlarged metacarpophalangeal and interphalangeal joints, and short stature [10]. Reported associations include corneal changes and osteoporosis [11, 12]. Characteristic imaging findings will be generalized platyspondyly and epiphyseal involvement, with enlargement of both ends of the short tubular bones of the hands [13].

Although radiologic examination has high accuracy in the diagnosis of PPD, the definitive diagnosis is established with identification of the characteristic radiological findings and biallelic pathogenic variants in WISP3 on molecular genetic testing [14].

Treatment is mainly conservative. Physical therapy may help preserve joint mobility. Immobilization (e.g., casting) should be avoided. Total replacement arthroplasty of the hips for secondary degenerative changes may be required at an early age and genetic studies need to be done for family counseling.

## 6. Conclusion

Genetic musculoskeletal disorders should be remembered among childhood rheumatic problems so that inappropriate therapy can be prevented while appropriate therapy could be started on time and genetic counseling can be offered to the family for the next generations.

## Abbreviations

PPD: Progressive pseudorheumatoid dysplasia
SEDT-PA: Spondyloepiphyseal dysplasia tarda with progressive arthropathy.
